# Bone scaffolds loaded with siRNA-*Semaphorin4d* for the treatment of osteoporosis related bone defects

**DOI:** 10.1038/srep26925

**Published:** 2016-06-02

**Authors:** Yufeng Zhang, Lingfei Wei, Richard J. Miron, Bin Shi, Zhuan Bian

**Affiliations:** 1 State Key Laboratory Breeding Base of Basic Science of Stomatology (Hubei-MOST) and Key Laboratory of Oral Biomedicine Ministry of Education, School and Hospital of Stomatology, Wuhan University, People’s Republic of China; 2 Department of Dental Implantology, School and Hospital of Stomatology, Wuhan University, People’s Republic of China; 3 Department of Restorative, Preventive and Pediatric Dentistry, University of Bern, Switzerland; 4 Department of Periodontology, Nova Southeastern University, Fort Lauderdale, Florida, USA

## Abstract

Osteoporosis is a prominent disorder affecting over 200 million people worldwide. Recently, semaphorins have been implicated in the cell-cell communication between osteoclasts and osteoblasts and have been associated with the progression of osteoporosis. Previously, we demonstrated that knockdown of semaphorin4d (Sema4d) using siRNA delivered with a bone-targeting system prevented bone loss in an osteoporotic animal model. Here, we used this bone-specific technology containing siRNA-*Sema4d* and fabricated a PLLA scaffold capable of enhancing bone repair following fracture. We investigated the ability of the implant to release siRNA-*Sema4d* into the surrounding tissues over time and to influence new bone formation in a 3 mm femur osteoporotic defect model in ovariectomized rats. Delivery of the bone-targeting system released from PLLA scaffolds began 2 hours post-implantation, peaked at 1 day, and was sustained over a 21 day period. μCT analysis demonstrated a significantly higher bone volume/total volume bone mineral density and number of osteoblasts in the rats that were transplanted with scaffolds loaded with siRNA-*Sema4d*. These results confirm the specific role of *Sema4d* in bone remodeling and demonstrate that significant increases in the speed and quality of new bone formation occur when siRNA-*Sema4d* is delivered via a PLLA scaffold.

Osteoporosis is a global healthcare issue with an increasing socio-economic burden. It is caused by an imbalance between bone-forming osteoblasts and bone-resorbing osteoclasts^1^,^2^. Over 200 million people are affected worldwide^3^, with the majority of patients being white or Asian women over 65 years old^4^. For decades, research has shown that osteoporotic patients demonstrate reduced healing after bone injury^5^. Fractures are more common, and their healing potential is drastically reduced^5^. The disease is caused by hyper-activity of osteoclasts, which affects the bone remodeling cycle and limits the ability of the incoming bone-forming osteoblasts to lay new bone matrix^2^,^6^,^7^,^8^,^9^,^10^.

At present, the two major pharmacological approaches for the treatment of osteoporosis are as follows: stimulation of bone formation via anabolic agents (such as parathyroid hormone) or prevention of bone resorption via anti-resorptives (such as bisphosphonates, calcitonin, raloxifene, and estrogen replacement therapy)^11^. Semaphorins have recently been targeted as molecules with osteoporosis treatment potential. They are directly implicated in the cell-cell communication between osteoclasts and osteoblasts and may be a novel target for the treatment of osteoporosis^12^,^13^,^14^,^15^,^16^,^17^,^18^,^19^,^20^,^21^. Furthermore, the overexpression of semaphorin4d (Sema4d) in bone tissues has been associated with osteoporosis in an animal model^22^. A *Sema4d* knockout animal model recently demonstrated an increase in bone thickness and density, further implicating Sema4d in the bone remodeling cycle.

Previously, we have developed a site-specific bone-targeting drug delivery system consisting of polymeric nanoparticles containing an siRNA-mediated gene knockdown system for *Sema4d*^23^. This system specifically targets native bone and releases siRNA-*Sema4d* onto bone surfaces occupied by osteoclasts. Weekly injections of this system significantly improved bone formation in both an early and late phase osteoporotic animal model by re-balancing the bone remodeling cycle^23^.

While most of the current osteoporosis research focuses on fracture prevention using a variety of pharmacological agents, the treatment of osteoporosis-related defects following fracture has not been as well studied. Therefore, in this study, we fabricated a specific bone replacement material from poly-L-lactic acid (PLLA) scaffolds to promote bone formation in an osteoporotic phenotype and studied this in 3 mm femur defects in ovariectomized (OVX) rats. The PLLA scaffolds were then loaded with a bone-specific targeting system that contained siRNA-*Sema4d* to improve bone remodeling, and new bone formation was investigated.

## Materials and Methods

### Preparation and characterization of PLLA

Four groups were used for all animal experiments: 1) drilled control, 2) PLLA alone, 3) PLLA-(Asp8-(STR-R8)) and 4) PLLA-(Asp8-(STR-R8)-siRNA*-Sema4d*. Porous PLLA scaffolds were prepared using a previously described freeze-drying method^24^. Briefly, 1g PLLA (Sigma #765112; Sigma-Aldrich, St. Louis, USA) was dispersed in 10 ml dioxane (Sinopharm Chemical Reagent Co., Ltd, Shanghai, China) under vigorous stirring until completely dissolved. Then, the mixture was rapidly transferred to a freezer at −35 °C overnight to solidify the solvent and induce solid–liquid phase separation. This solidified mixture was then maintained at −80 °C for 2 h and subsequently transferred into a freeze-drying vessel (Christ Beta 1–15, Germany) for 48 h until dried. Scaffolds were then sterilized and lyophilized. The Asp8-(STR-R8)-siRNA-*Sema4d* (covalently linked) bone targeting system was fabricated as previously described^23^. Briefly, the packaging of siRNA (GenePharma, Suzhou, China) was performed according to a protocol adapted from DNA transfection experiments^25^. Asp8-Stearyl-R8 (1.5 μl) (ChinaPeptides Co., Ltd, Shanghai, China) was diluted in 50 μl of unsupplemented Neurobasal medium (Gibco^®^, USA) and combined with 10 pmol of siRNA in 50 μL of unsupplemented Neurobasal medium. The solution was incubated for 5 min at room temperature. The PLLA (0.01 g) was then incubated with 1 ml of Asp8-(STR-R8)-siRNA*-Sema4d* solution or Asp8-(STR-R8) solution (5 OD/ml) at 4 °C overnight to allow complete infiltration. These complexes were then frozen by immersion at −80 °C for 2 h and subsequently lyophilized.

### Animals and surgical procedures

Mature female Wistar rats (12 weeks old, mean body weight 250 g) were purchased and used for this study. All handling and surgical procedures were approved by the Ethics Committee for Animal Research, Wuhan University, China. The methods were carried out in “accordance” with the approved guidelines. Animals received food and water ad libitum and were housed at a constant temperature of 22 °C. For surgery, the animals were place under general anesthesia using an intraperitoneal injection of chloral hydrate (Sinopharm Chemical Reagent Co., Ltd, Shanghai, China, 10%, 4 ml/kg body weight), and all operations were performed under sterile conditions with a minimally invasive surgical technique. Postoperatively, penicillin (40,000 IU/ml, 1 ml/kg) was injected each day for 3 days. There were no signs of inflammation or other notable anomalies.

### Osteoporosis model

The animals were acclimatized to the new laboratory surroundings for one week. The osteoporotic animal model was established using a bilateral ovariectomy (OVX)^26^. Briefly, under general anesthesia, the rats received 10 mm linear incisions bilaterally in the lumbar skin. Both ovaries were gently removed. The tissue layers were then repositioned and sutured. Following surgery, buprenorphine (0.05 mg/kg) was administered by subcutaneous injection for pain management.

### Femoral defect model

Standardized 3 mm femoral cylindrical defects were created in ovariectomized animals 2 months after the bilateral removal of the ovaries. The osteoporosis model should have been established at this time point according to previous studies^27^,^28^. A distal femoral epiphysis linear skin incision (approximately 1 cm) was performed bilaterally. The femoral condyles were then exposed by blunt dissection of the muscles surrounding the femurs. A 2.8 mm diameter reamer was used to create a 3.0 mm diameter anteroposterior bicortical channel perpendicular to the shaft axis (Supplemental Fig. 1A (reprint with permission from Cheng *et al*.^27^)). The bur was irrigated at a slow speed (800 rpm) and rinsed with saline solution to avoid thermal necrosis of the cells and tissues. The bone fragments were washed out of the cavity using the saline solution. Scaffolds (3 mm in diameter by 5 mm in thickness) were gently placed bilaterally according to a randomized group allocation. The incisions were then closed. Buprenorphine (0.05 mg/kg) was administered by subcutaneous injection for postoperative pain management.

### Pharmacokinetics study

PLLA scaffolds were cut using custom trays (3 mm in diameter and 5 mm in thickness), gently loaded with rhodamine-labeled Asp8-(STR-R8) and placed within the femurs. Samples were collected at 0 hours, 12 hours, 1 day, 3 days, 7 days, 14 days and 21 days post-operation to assess the duration and location of drug distribution. The fluorescence signal was detected using a Xenogen IVIS imaging system (Xenogen, Alameda, Ca, USA) as previously described^23^.

### Bone regeneration

To study bone regeneration in the osteoporotic femoral defect, the rats were divided into four groups: 1) drilled control, 2) filled with PLLA, 3) filled with PLLA-(Asp8-(STR-R8)) and 4) filled with PLLA-(Asp8-(STR-R8)-siRNA*-Sema4d*) (n = 15 in all groups). Rats were sacrificed at 2, 4 and 8 weeks after femoral surgery. The defect, size, shape and location are presented in supplemental Fig. 1. The rats were divided into four groups with 6 defects per group per time point.

### Micro-CT (μCT) analysis of osteogenesis in the femoral defect

After harvesting at each time point, the femurs were removed intact and fixed in 4% freshly prepared formaldehyde for 24 h at 4 °C. The μCT imaging system (μCT50, Scanco Medical, Brüttisellen, Switzerland) was used to evaluate the osteogenesis within the defect region. The study parameters used were previously reported^26^,^29^,^30^,^31^. The scanning parameters, reconstructed parameters and analysis parameters were entered into the μCT scanner. A consistent volume of interest (VOI) was located in the central 2.5-mm-diameter region of the 3-mm-diameter defect. The middle third (by length) was used to evaluate the level of bone regeneration. Mineralized bone tissue was differentially segmented with a fixed low threshold (value = 184). The bone volume fractions (BV/TV), trabecular thickness (Tb.Th) and tissue mineral density were automatically collected and analyzed using the μCT software as previously described^26^,^29^,^30^,^31^. Representative sections were taken from the vertical view, and a representative cube of VOI was acquired by 3D reconstruction.

### Histological observation and analysis

Following μCT scanning, femurs were removed and decalcified in 10% EDTA. The solution was changed twice weekly for 3 weeks. The samples were embedded in paraffin with the long axis parallel to the base plane. Serial sections (5 μm) were cut and mounted on polylysine-coated slides. The samples were analyzed using H&E staining and tartrate-resistant acid phosphatase (TRAP) staining (Sigma #387A; Sigma-Aldrich, St. Louis, USA) in accordance with the manufacturer’s protocol. The expression of SEMA4D and osteocalcin (BGLAP) were detected according to the following immunohistochemical procedure. The samples were deparaffinized, rehydrated and washed. The sections were then processed for antigen retrieval by using trypsin and then incubated with 0.3% hydrogen peroxide for 20 min followed by incubation with normal goat serum. The sections were incubated with a primary antibody specific for SEMA4D (1:250; 14422-1-AP, Proteintech. Inc., US) or BGLAP (1:200; BA1677-1, Boster, China) for 2 h at 37 °C. The sections were then incubated using the SP 9000 immunohistochemical kit (Zhongshan Biotechnology Co., Ltd, China) and visualized with 3, 3-diaminobenzidine tetrahydrochloride (DAB) (Zhongshan Biotechnology Co., Ltd, China). The sections were counterstained with hematoxylin. TRAP-positive osteoclasts were stained red and were identified by the presence of three or more nuclei. Each defect was assayed in triplicate for each of the 6 animals per group at the 3 time points (54 samples per group).

### Statistical analysis

Statistical analyses were performed using a one-way ANOVA and the Student-Newman-Keuls test. Results were considered statistically significant at p < 0.05. All data are expressed as the mean ± standard deviation (SD).

## Results

### Sustained and localized release *in vivo*

**Figure 1:**
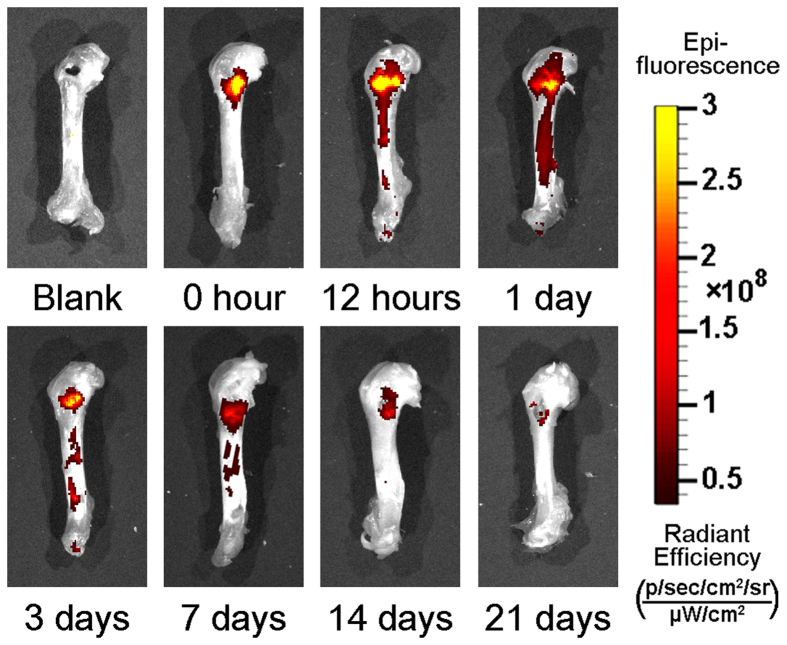
The kinetics of Asp8-(STR-R8) release/distribution in femurs over 21 days. The epi-fluorescence indicated sustained and localized drug distribution restricted to the defect and the adjacent bone tissue and peaking at 1 day post-implantation.

Images of a representative femur filled with PLLA scaffold incorporating rhodamine-labeled Asp8-(STR-R8) depict the kinetics of the sustained and localized drug distribution for a period of 21 days (Fig. 1). The release of (measurable) Asp8-(STR-R8) was used as a surrogate marker for the release of siRNA-*Sema4d*. The rhodamine-labeled Asp8-(STR-R8) was released from PLLA scaffolds over time but restricted to the defect and adjacent bone tissue. The highest labeling was observed at 1 day post-implantation of the scaffold, and labeling was maintained (albeit in low quantities) for 21 days (Fig. 1).

### μCT analysis of osteogenesis in the femoral defect

**Figure 2:**
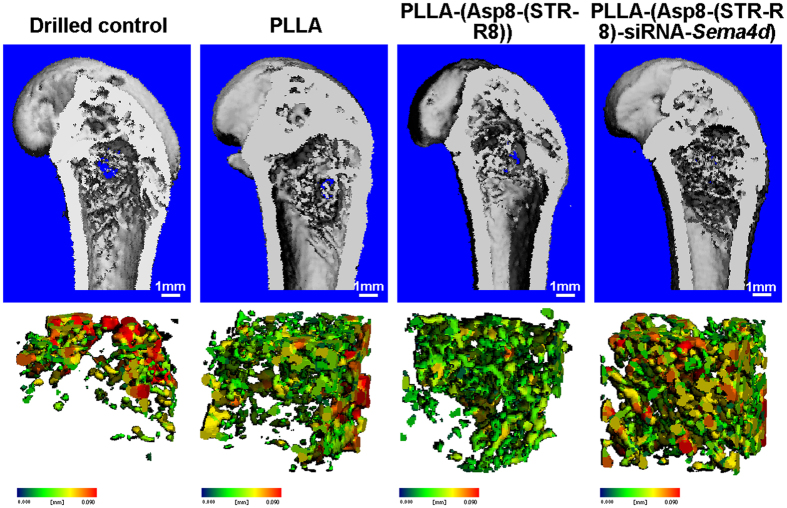
Representative images of overall bone regeneration using 3D reconstruction at 8 weeks post-implantation. The siRNA-*Sema4d* treated group had significantly increased mineralization in trabecular bone compared to the control, PLLA alone and PLLA-(Asp8-(STR-R8)-siRNA*-Sema4d* loaded defects. The bottom row represents the 3-dimensional reconstruction of the bone defects from the micro-CT analysis, demonstrating higher bone density for the group treated with siRNA-*Sema4d.*

**Figure 3:**
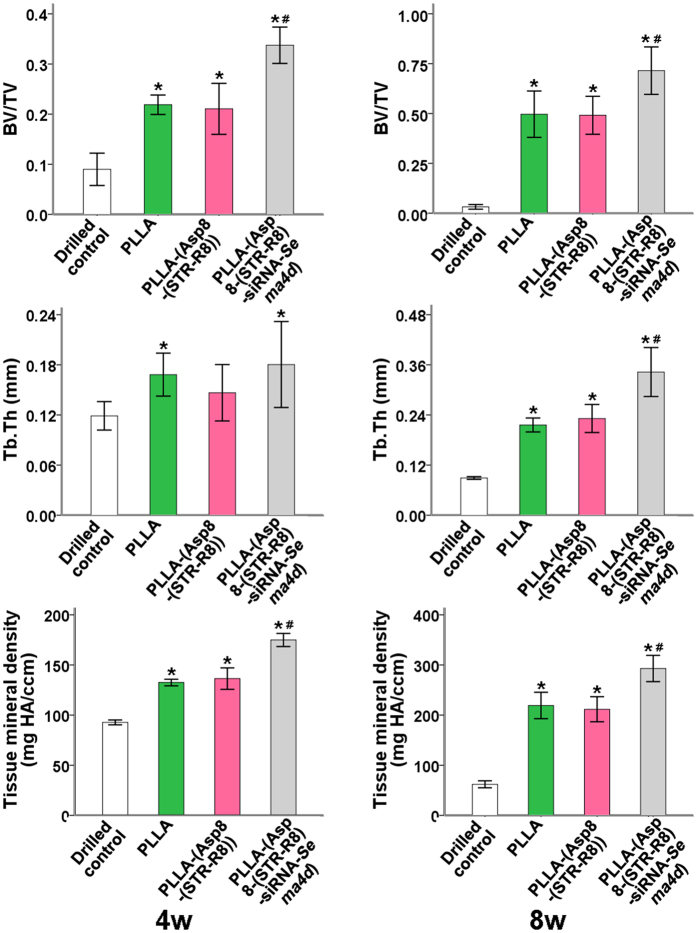
Statistical analysis of bone volume/total volume (BV/TV), trabecular thickness and tissue mineral density in the bone defect as assessed by μCT. All data are shown as the mean ± SD. P < 0.05. *Compared to drilled blank control; **significantly higher than all other groups.

Representative images of bone regeneration in the femoral defect, performed by 3D reconstruction for each group, are shown in Fig. 2. A minimal amount of new bone formation was found in the unfilled defects at all time points (Fig. 2). The three defects filled with scaffolds demonstrated improved bone formation and mineralization compared to the unfilled defects. The siRNA-*Sema4d* treated group regenerated the highest amounts of mineralized bone (Fig. 2). Quantitative analyses demonstrated that the bone volume, trabecular thickness and tissue mineral density of the siRNA-*Sema4d* treated group were significantly higher than those of the PLLA filled group at 8 weeks post-operation (Fig. 3). The unfilled group had the lowest values of all treatment groups at 4 and 8 weeks post-operation (Fig. 3).

### Histological and immunohistochemical analysis

**Figure 4:**
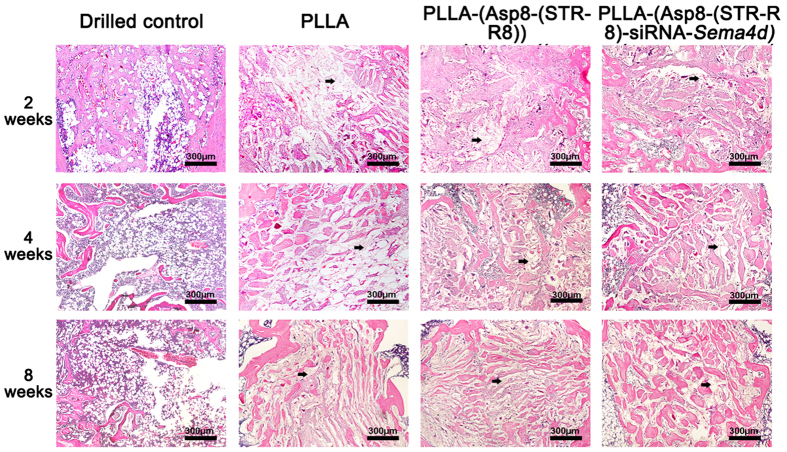
Representative sections with H&E staining for all treatment groups.

Representative images of H&E staining of the femoral defect are shown in Fig. 4. At 2 weeks post-implantation, bone regeneration was observed at the periphery of the defect sites in all groups in which the defects were filled with scaffolds. There were no significant differences between groups and only a small amount of new bone regeneration had occurred (Fig. 4). At 4 weeks post-implantation, the unfilled group demonstrated little to no new bone formation. Bone formation had not improved further at 8 weeks post-surgery (Fig. 4). The scaffold groups had a significantly larger bone area than did the controls at both 4 and 8 weeks (Fig. 4). The scaffolds that were pre-loaded with siRNA-*Sema4d* further supported new bone formation compared to all other treatment modalities at 4 and 8 weeks.

**Figure 5:**
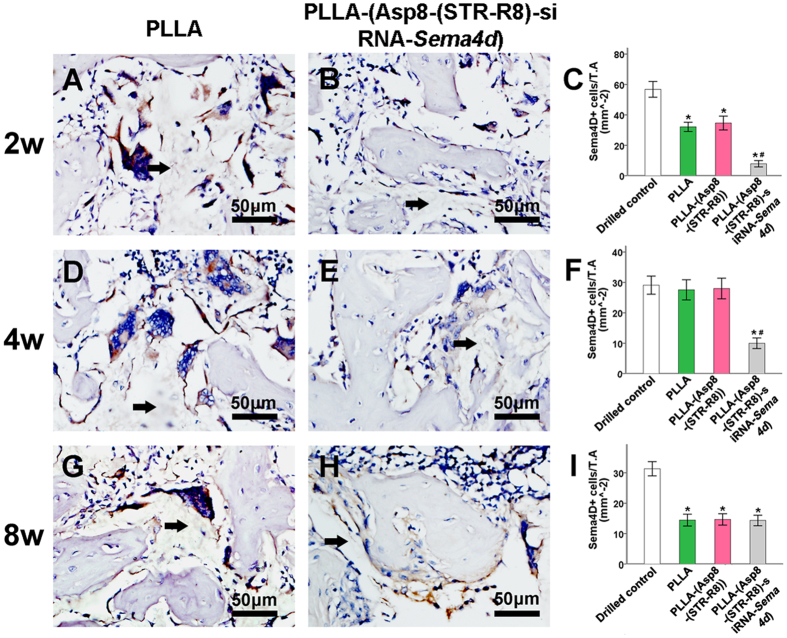
Representative sections stained for *Sema4d*-positive cells revealed the success of the sustained knockdown of *Sema4d*. A significant reduction in the number of *Sema4d* positive cells in the PLLA-(Asp8-(STR-R8)-siRNA*-Sema4d*) filled group was observed at 2 and 4 weeks post-implantation compared to the other groups. By 8 weeks post-implantation, no differences were observed between the groups that received the scaffolds. Control unfilled scaffolds had significantly more Sema4d-positive cells than all other groups. All data are shown as the mean ± SD. P < 0.05. *Compared to drilled blank control; **significantly higher than all other groups.

**Figure 6:**
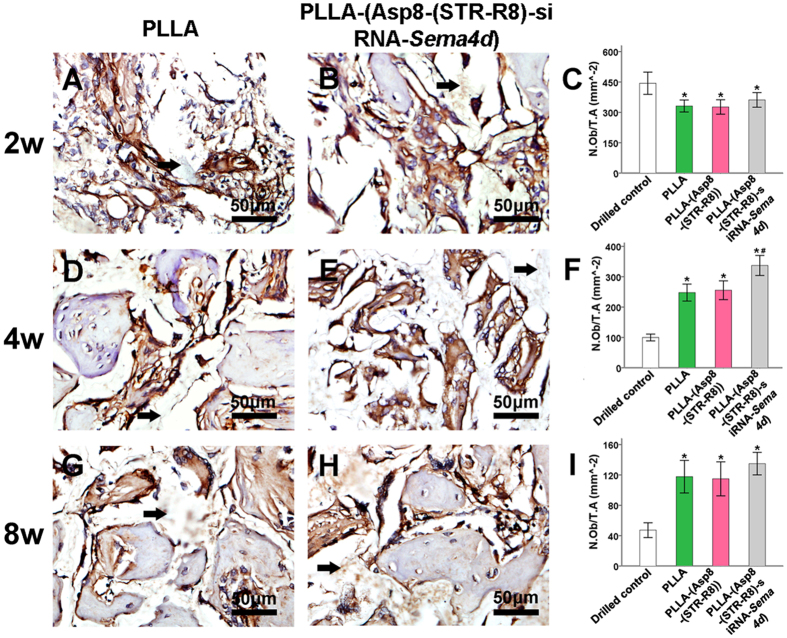
Representative images of BGLAP immunohistochemical staining revealed osteoblast-related bone regeneration in the filled bone defect. The statistical analysis indicated increased numbers of activated osteoblasts at 4 weeks post-operation in the PLLA-(Asp8-(STR-R8)-siRNA*-Sema4d*) filled group than with all other modalities. All data are shown as the mean ± SD. P < 0.05. *compared to drilled blank control; **compared to all other groups.

**Figure 7:**
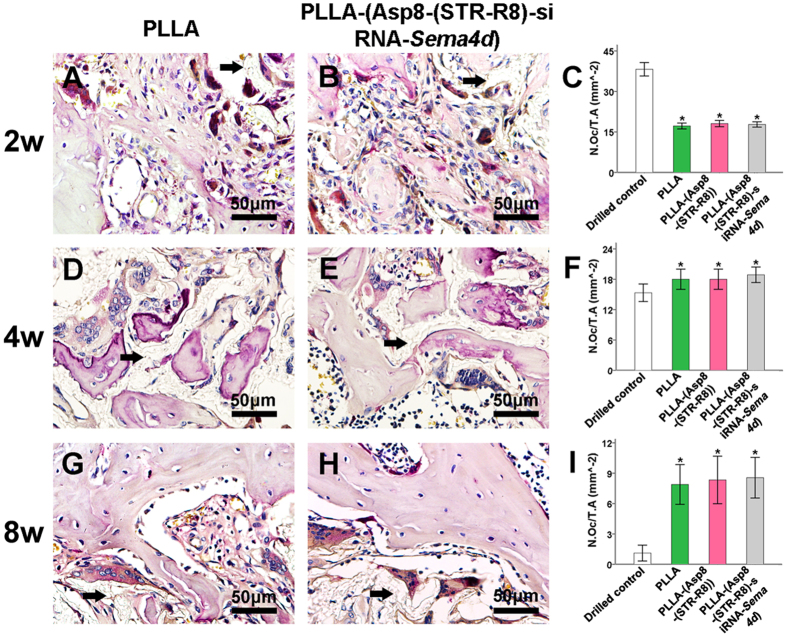
Representative sections revealed the bone remodeling process by TRAP positive osteoclasts during the pre-arranged observation period at 2, 4 and 8 weeks post-implantation. The statistical analysis indicated no difference in TRAP staining between the three filled defects at any of the tested time points. All data are shown as the mean ± SD. P < 0.05. *compared to drilled blank control).

Quantitative analysis was used to determine the number of Sema4d-stained cells (Fig. 5), the number of osteoblasts (Fig. 6) and the number of osteoclasts (Fig. 7). Staining of *Sema4d* positive cells demonstrated a 3-fold decrease in such cells at 2, 4 and 8 weeks in scaffolds that contained siRNA-*Sema4d*. This result confirmed the functioning of the *in vivo* knockdown system (Fig. 5). The number of osteoblasts was counted in the histological sections, which were also stained with BGLAP (Fig. 6). No difference in osteoblast number was observed at 2 weeks between all groups implanted with scaffolds. However, the scaffolds with siRNA-*Sema4d* led to significantly higher number of osteoblasts at 4 weeks than did the control scaffolds and returned to baseline PLLA control values by 8 weeks (Fig. 6). TRAP-positive osteoclasts were observed in all four groups around newly formed bone, with no differences among the three implanted scaffold groups (Fig. 7).

## Discussion

Recently, semaphorins have been targeted as molecules that are directly implicated in the cell-cell cross-talk between osteoclasts and osteoblasts^12^,^13^,^14^,^15^,^16^,^17^,^18^,^19^,^20^,^21^. They were originally identified as axon-guidance molecules but have since also been demonstrated to have important roles outside the nervous system. They play key roles in cell migration, cell-cell communication, tissue development and angiogenesis^32^,^33^,^34^,^35^,^36^,^37^. Their specific targeted delivery is important because of their widespread activity in the nervous system and other tissues. We previously developed a site specific targeting system with D-Asp8^23^. When intravenously injected into rats (over 4 weeks), we were able to locate and target bone with little to no delivery to surrounding tissues^23^. In the present study, we developed a scaffold carrying siRNA-*Sema4d* and investigated its release into bone tissues over time. In the past few years, siRNA has been investigated for the treatment of various diseases because of its ability to silence various genes responsible for pathology^38^,^39^,^40^,^41^. At 1 day post-implantation, the release of the vector had peaked and then gradually decreased over time (Fig. 1). The bone targeting system was expressed in the shaft of the femur. The delivery of this targeting system to bony tissues near the fracture site is a desirable feature because the development of osteoporosis leads to a reduction in bone specifically in the trabecular regions. The delivery system has previously been shown to target native bone undergoing bone remodeling. In the present study, we demonstrated that local implantation of this system into tissues with low bone density (an osteoporotic animal model) may improve the regeneration of bone following fracture/bone defects. Therefore, local implantation of a scaffold system that is both a bone replacement material and an osteopromotive agent may result in improved fracture healing in patients.

It has previously been reported from studies of knockout animals that the role of *Sema4d* in osteoclast activity is mediated by its receptor, Plexin-B1, which is expressed by osteoblasts^16^,^42^. There is now increasing evidence from pre-clinical studies that Sema4d is expressed at a higher level in osteoporotic animals^22^. Therefore, *Sema4d* is an important and viable target for gene silencing. In our previous study, we demonstrated that the knockdown of *Sema4d* in osteoclasts, which were co-cultured with bone marrow stromal cells, led to an increase in alkaline phosphatase activity. It also increased the mineralization potential and the upregulation of the genes encoding collagen 1 and osteocalcin as assessed by real-time PCR^23^. The present study further supports these results by demonstrating that the local implantation of this scaffold system increased tissue mineral density and BV/TV (Figs 2, 3, 4). The staining of Sema4d-positive cells was significantly decreased (over 3-fold) compared to blank or scaffold controls (Fig. 5). The scaffold system had little to no effect on the number of osteoclasts following transfection with siRNA-*Sema4d* and thereby did not halt the bone remodeling cycle (commonly reported during bisphosphonate use). One limitation of this study is that it does not focus on the activity of osteoclasts. Further research is needed to determine the potential effects of this system on the resorption properties of osteoclasts *in vitro*.

## Conclusions

The PLLA scaffold containing siRNA-*Sema4d* improved new bone formation in an osteoporotic animal model. The knockdown of *Sema4d* in osteoclasts did not affect the number of osteoclasts on the bone surface. However, the number of osteoblasts significantly increased, suggesting that Sema4d has a role in the cross-talk between osteoclasts and osteoblasts. The field of biomaterials faces many challenges in meeting the globally increasing demand for the repair of osteoporosis related fractures. We demonstrate that the site specific delivery of a bone-targeted gene knockdown system significantly improved new bone formation in osteoporosis related bone defects.

## Additional Information

**How to cite this article**: Zhang, Y. *et al*. Bone scaffolds loaded with siRNA-*Semaphorin4d* for the treatment of osteoporosis related bone defects. *Sci. Rep.* **6**, 26925; doi: 10.1038/srep26925 (2016).

## Supplementary Material

Supplementary Information
