# Supplementary material for: Bone scaffolds loaded with siRNA-Semaphorin4d for the treatment of osteoporosis related bone defects
Source: Sci Rep. 2016 Jun 2;6:26925. doi: 10.1038/srep26925 (PMC4890584; doi:10.1038/srep26925)
Supplement: Supplementary Information [file srep26925-s1.doc]

**Bone scaffolds loaded with siRNA-*Semaphorin4d* for the treatment of osteoporosis related bone defects**

Yufeng Zhang^1,2^, Lingfei Wei^1^, Richard J. Miron^1,3,4^, Bin Shi^1,2^ and Zhuan Bian^1^ *

**Supplemental Files:**

Supplemental Figure 1: Femur defect drilling was performed in the OVX rats, followed by implantation of the scaffolds. Schematic presentation of the dimension and position of the femur defect using μCT images (**b**, **c**) (reprint with permission from Cheng et al. 2013).

**
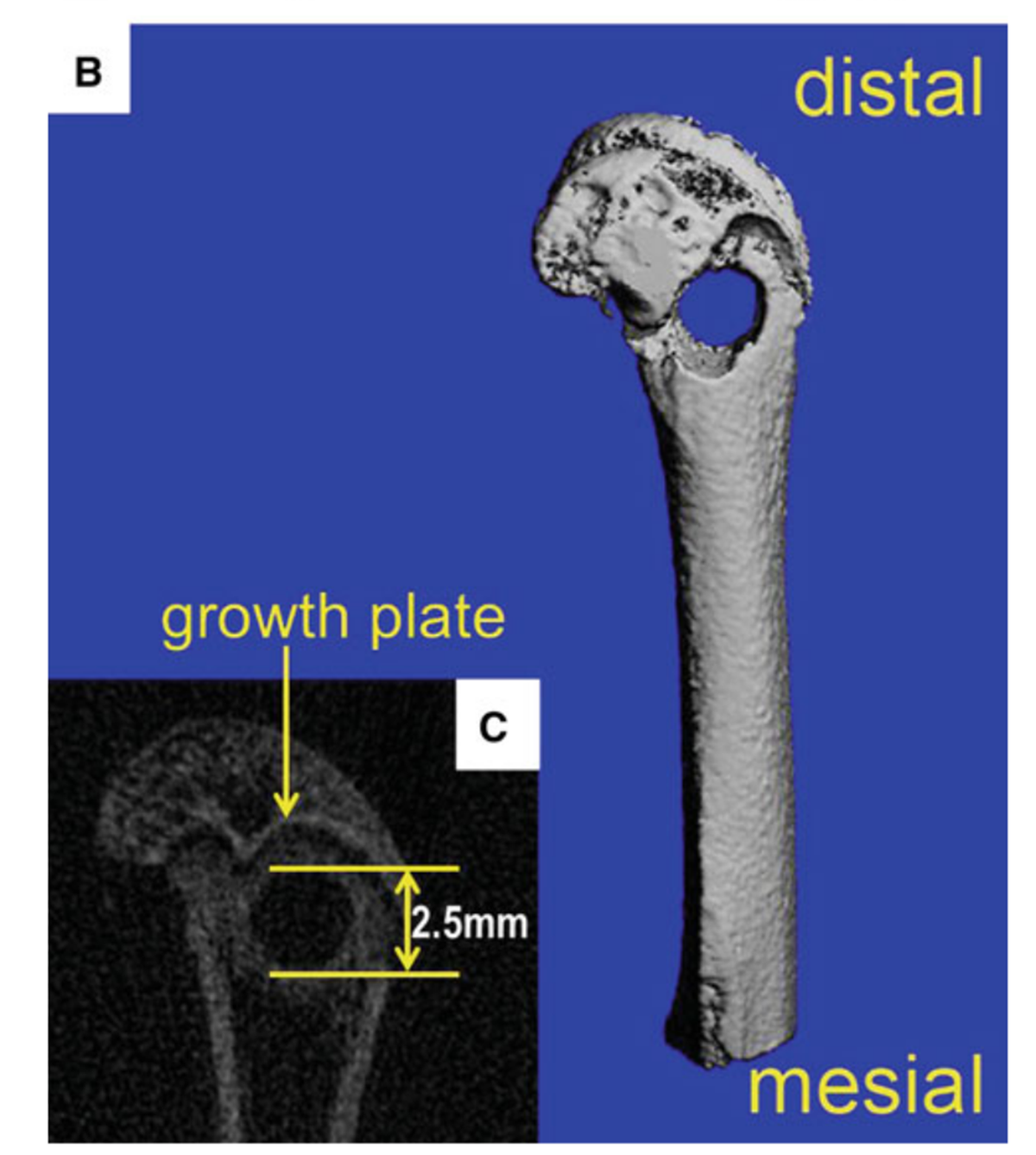
**
